# ICT Self-Efficacy, Organizational Support, Attitudes, and the Use of Blended Learning: An Exploratory Study Based on English Teachers in Basic Education

**DOI:** 10.3389/fpsyg.2022.941535

**Published:** 2022-06-28

**Authors:** Long Ye, Manteng Kuang, Song Liu

**Affiliations:** ^1^School of Foreign Studies, Shaoguan University, Shaoguan, China; ^2^College of English and Foreign Studies, Beijing Foreign Studies University, Beijing, China; ^3^College of Foreign Studies, Hunan University of Finance and Economics, Changsha, China

**Keywords:** blended learning, organizational support, ICT self-efficacy, English teachers, basic education

## Abstract

The study aims to build a model that predicts the behavior of the use of blended learning by English teachers of basic education in China in the environment of repeated lockdowns during the COVID-19 pandemic. It examines the relationships between ICT self-efficacy, organizational support for blended learning, attitudes toward blended learning, and the use of blended learning. Data were collected from 562 teachers using a survey questionnaire. Employing partial least squares structural equation modeling (PLS-SEM), a hypothesized model was tested for path coefficients and predictive power. This study found that ICT self-efficacy and organizational support for blended learning are sound predictors of teachers' use of blended learning and that the former appeared to be a stronger determinant. In addition, attitudes toward blended learning can directly influence teachers' use of blended learning and mediate the relationship between ICT efficacy and the use of blended learning and between organizational support for blended learning and the use of blended learning. These three variables account for 54.7% of the variance in teachers' use of blended learning.

## Introduction

Blended learning, the integration of face-to-face learning and online learning experiences (Garrison and Kanuka, 2004), has long been a development trend of teaching/learning methods in higher education. Its effectiveness in higher education has been well established (López-Pérez et al., 2011; Bernard et al., 2014; Simonova, 2019). The use of the online teaching method requires teachers who want to adopt the blended learning approach to have certain information/internet and communication technology (ITC) competencies and experience. Although ICT makes interactive and self-directed learning more convenient (Mishna et al., 2012) and the benefits of ICT in education have been recognized (Vichitvejpaisal et al., 2001; Higgins, 2003; Allan and Will, 2008), many teachers are reluctant to integrate ICT into classroom teaching (Cuban, 2001; Varank and Tozoglu, 2006; Becta, 2008), largely due to a lack of confidence in the use of ICT, and this phenomenon is more prevalent at the basic education stage (Jimoyiannis and Komis, 2007; Yang and Huang, 2008). In addition, the implementation of a blended learning approach requires substantial support from the organization, which includes organizational leadership, policy, planning, and resources from the school (Garrison and Kanuka, 2004). Meeting these requirements is relatively more difficult for basic education. For the above reasons, compared with higher education, blended learning is less used in basic education, and the relevant research results are relatively insufficient.

Due to the repeated lockdowns during the COVID-19 pandemic, in many regions of China, blended teaching has become almost the only option for teachers to keep up with teaching progress, whether at universities or in basic education. In the past 2 years, Chinese teachers have become accustomed to using different online platforms, such as DingTalk, Tencent Meeting, Zoom, and WeChat to complement their offline face-to-face teaching. This suggests that blended learning may become a routine for teachers in the post-pandemic period (Wong, 2020). The technology acceptance model (TAM) developed by Davis (1986, 1989) is currently the most popular theoretical model for research on blended learning. The intention to adopt blended learning and the perceived ease of use and usefulness of a specific blended learning system are the focuses of previous TAM-based research (Al-Azawei et al., 2016; Martín-García et al., 2019). However, under special circumstances of the epidemic, whether or not teachers think that a blended system is easy to use or useful, they have had to adopt blended learning for their students since it is their only option. Therefore, in the current environment, research on external variables in TAM, such as environmental support and self-efficacy, and actual blended teaching/learning behaviors, are more in line with the practical application.

Based on the above background, the present study attempts to explore the effects of ICT self-efficacy, organizational support, and attitudes toward blended learning on teachers' behavior in using blended learning methods during the COVID-19 pandemic. The results of this study are based on survey data of primary and lower secondary school English teachers in Guangdong Province, China. Two objectives are included in this study: to explore the relationships between teachers' ICT self-efficacy, organizational support, and attitudes toward blended learning and their behavior in using blended learning methods and to examine the mediating role of teachers' attitudes in the conceptual model proposed in this research.

## Literature Review

### Blended Learning

Garrison and Kanuka (2004) provided a simple definition of blended learning as “the thoughtful integration of classroom face-to-face learning experiences with online learning experiences.” Blended learning can be applied to different levels, such as subject, curriculum, activities, and institutional levels (Graham, 2010), and it may combine students and/or teachers both online and in the classroom with online learning activities and classroom activities (Osguthorpe and Graham, 2003). Blended learning has been regarded as an approach that integrates the advantages of both face-to-face learning and online learning and avoids the drawbacks of each method (Osguthorpe and Graham, 2003; Thorne, 2003). It has been indicated that blended learning is an effective educational approach in many studies (Means et al., 2005; Bernard et al., 2014; Qian et al., 2016; Vo et al., 2017) and compared with the traditional face-to-face approach, blended learning has been shown to be more effective in bringing about positive impacts on learning (Lu, 2021).

### ICT Self-Efficacy

Bandura (1986, p. 391) defined self-efficacy as “people's judgments of their capabilities to organize and execute courses of action required attaining designated types of performances.” It refers to individuals' perceived capabilities in specific areas and reflects what they believe they can do with the skills they possess (Bandura, 1997).

The definition of ICT self-efficacy is mainly based on computer self-efficacy, which refers to individuals' judgments of their own capabilities to accomplish a task using computers. For teachers, computer self-efficacy refers to their perceived capabilities in applying computers for teaching aims (Scherer and Siddiq, 2015). With the advancement of internet technology, ICT has gradually been adopted by an increasing number of teachers for teaching purposes, so people's attention to computer self-efficacy has evolved into ICT self-efficacy. ICT self-efficacy is confidence in teachers' competencies in using ICT for teaching and learning activities (Siddiq et al., 2016; Moreira-Fontán et al., 2019). Wang and Zhao (2021) defined ICT self-efficacy as teachers' perceived judgments of their capabilities to master and use technologies, such as multimedia, computers, and the internet, to achieve instructional purposes. Papastergiou (2010) states that ICT self-efficacy is the combination of computer self-efficacy and internet self-efficacy. Previous research findings have shown that ICT self-efficacy plays a crucial role in both students' learning processes and teachers' instructional processes (Aesaert and van Braak, 2014; Moreira-Fontán et al., 2019).

### Organizational Support

Organizational support originates from organizational support theory (Eisenberger et al., 1986, 1997). Eisenberger et al. (1986) conceptualized perceived organizational support as employees' perceptions of the extent to which their organization thinks highly of their contributions. Previous studies show that antecedents of perceived organizational support include fairness (Shore and Shore, 1995), supervisor support (Yoon and Lim, 1999; Rhoades et al., 2001), organizational rewards (Rhoades and Eisenberger, 2002), and job conditions, such as job training (Wayne et al., 1997) and role stressors (Lazarus and Folkman, 1984).

Research has shown that the level of perceived organizational support is positively related to employees' performance (George and Brief, 1992) and job involvement (O'Driscoll and Randall, 1999) and negatively related to their level of stress (Viswesvaran et al., 1999).

## Research Hypotheses

### Attitude Toward Blended Learning (ABL) and the Use of Blended Learning (UBL)

Ajzen and Fishbein (1980) model of the theory of reasoned action (TRA) provides a critical conceptual model for explaining human behavior. Based on the TRA, Davis (1989) developed the technology acceptance model (TAM) and introduced a new theoretical model to explain the relationship between attitudes toward behavioral intention and behavior. In both models, attitude is an antecedent of actual behavior mediated by behavioral intention. The effectiveness of the TAM in the interpretation of the acceptance of new technologies, especially ICTs, has been well established (Dillon and Morris, 1996; Silva and Dias, 2007; Yuen and Ma, 2008; Guner and Acarturk, 2018). From the above research, the following hypothesis was formulated.

H1: ABL has a significant influence on UBL.

### ICT Self-Efficacy (ICTSE), ABL, and UBL

Previous studies indicate that ICT self-efficacy is a reliable predictor of ICT competencies (Barbeite and Weiss, 2004; Sam et al., 2005; Aesaert and van Braak, 2014) and has been proven positively related to the adaptation to technological changes and new environments (Sam et al., 2005; Papastergiou, 2010). When applying ICT to the teaching process, teachers with greater ICT self-efficacy have stronger positive emotions (Moreira-Fontán et al., 2019). TAM-based research normally treats ICT/computer self-efficacy as an external variable, and research results consistently show that it is significantly correlated with attitude and behavior (Scott and Walczak, 2009; Teo, 2009; Van Acker et al., 2011). There is also research evidence that the success of blended learning is largely determined by ICT capabilities (Swan, 2001; Garrison and Anderson, 2003). Therefore, the following hypotheses were formulated.

H2: ICTSE has a significant influence on ABL.

H3: ICTSE has a significant influence on the UBL.

### Organizational Support of Blended Learning (OSBL), ABL, and UBL

The research of Bogler and Nir (2012) has shown that teachers' perception of their school support is related to their intrinsic and extrinsic job satisfaction. There are also research results showing that teachers' perceived organizational support is correlated with their attitudes toward their job and their job performance (Xu and Yang, 2018). Nayir (2012) points out that teachers feeling more supported by their school tend to contribute more to the objectives of their school, so the perception of organizational support is crucial for them to internalize the organizational objectives. In the case of blended learning, the research results of Zhao and Song (2021) indicate that teachers expect organizational support, such as recognition and understanding, guidelines, incentive policy, funds, network conditions, training on educational technology, technical assistance, and experience sharing from their schools for blended learning implementation. From the above literature, the following hypotheses were proposed.

H4: OSBL has a significant influence on ABL.

H5: OSBL has a significant influence on the UBL.

### ABL as the Mediator

Attitude is used as a mediating variable in both TRA theory and TAM. In TRA theory, attitude normally precedes external variables and is followed by behavior intention as a predictor (Ajzen and Fishbein, 1980). Similarly, in the TAM, attitudes are also used as a predictor of behavioral intentions but will normally precede the usefulness and ease of use of the technology involved (Ajzen and Fishbein, 1980). Based on these two theories, numerous studies have shown that attitude is a sound mediating variable. Hence, we propose the following two hypotheses.

H6: The relationship between ICTSE and the UBL is mediated by ABL.

H7: The relationship between OSBL and the UBL is mediated by ABL.

## Methodology

### Research Design

**Figure 1:**
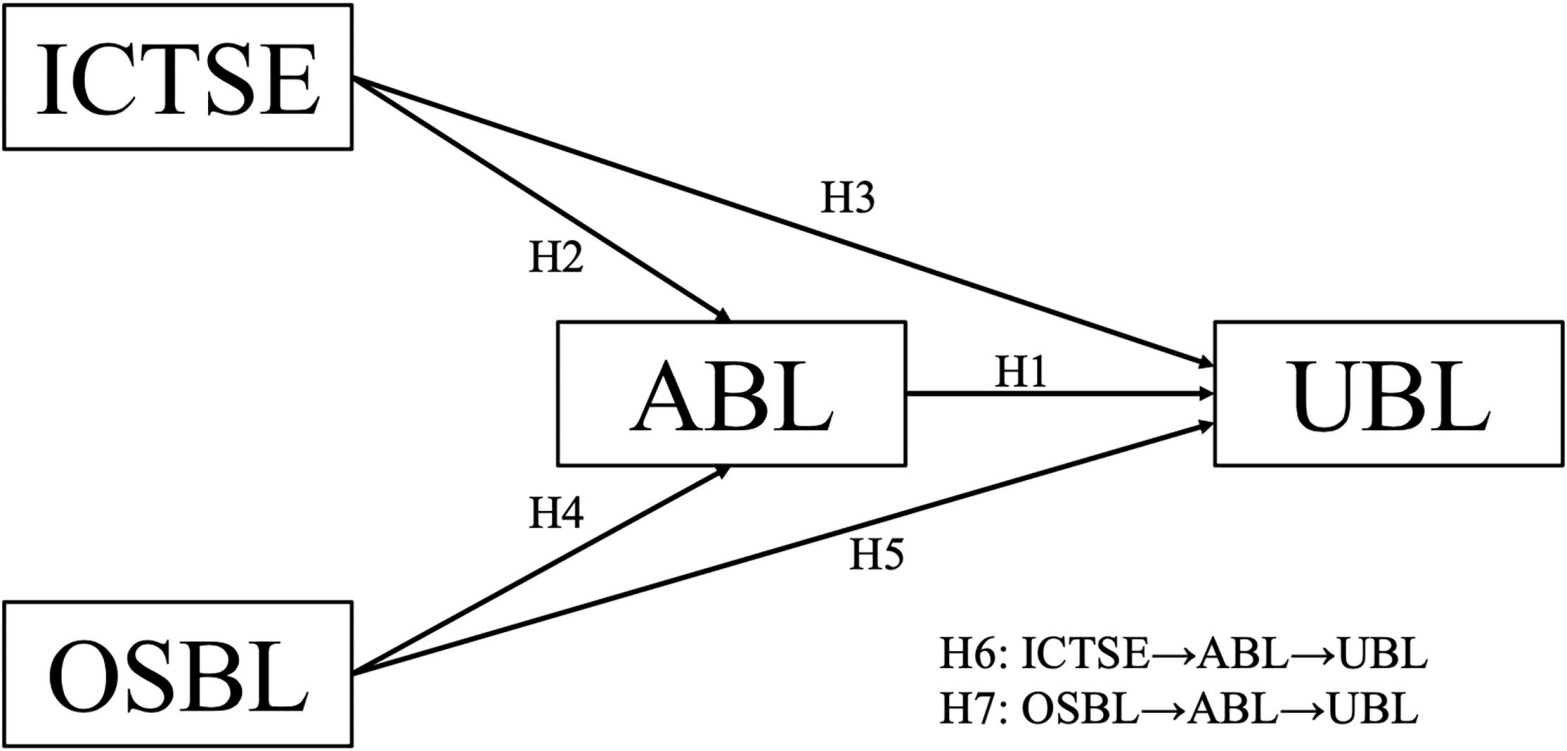
A research model for this study. ICTSE, ICT self-efficacy; OSBL, organizational support for blended learning; ABL, attitude toward blended learning; UBL, the use of blended learning.

Based on the hypotheses formulated, the present study developed a model that represents the relationships between the four variables, ICT self-efficacy, organizational support for blended learning, attitude toward blended learning, and the use of blended learning, with a structural equation modeling approach. A survey questionnaire consisting of questions on demographics and multiple items for each construct in the research model (Figure 1) was used for data collection.

### Research Participants

The participants in the present study were 562 English teachers of basic education in Guangdong Province, China. As shown in Table 1, the participants represent English teachers of various educational backgrounds, teaching experiences, and different school levels and genders. In terms of gender, 10.1% (51) of them are male, and 89.9% (505) are female. There were 223 (39.7%) primary school teachers and 339 (60.3%) lower secondary school teachers. Most teachers (514/91.5%) had a bachelor's degree as their final degree, 34 (6%) teachers had attended three-year colleges without a degree, several (12/2.1%) held a master's degree, and 2 (0.4%) teachers had no higher education background. In terms of teaching experience, there were 259 (46.1%) teachers with more than 20 years of teaching experience, 79 (14.1%) teachers with 16–20 years of teaching experience, 55 (9.8%) teachers with 11–15 years of teaching experience, 41 (7.3%) teachers with 5–10 years of teaching experience, and 128 (22.8%) teachers with <5 years of teaching experience.

**Table 1:** Demographic information of the participants (*N* = 562).

**Variable**	**Number**	**(%)**
**Gender**		
Male	57	10.1
Female	505	89.9
**School Level**		
Primary School	223	39.7
Lower Secondary School	339	60.3
**Education Background**		
Master's Degree	12	2.1
Bachelor's Degree	514	91.5
Three-year College without Degree	34	6
No higher education	2	0.4
**Teaching Experience**		
<5 Years	128	22.8
5–10 Years	41	7.3
11–15 Years	55	9.8
16–20 Years	79	14.1
>20 Years	259	46.1

### Instruments

A questionnaire was designed to measure the four constructs in the research model. There are two sections in the questionnaire: the first requires participants to provide demographic information, and the second includes 14 items on the four constructs in this study. Each item was measured on a five-point Likert scale, scoring from 1 (strongly disagree) to 5 (strongly agree). These items of statements were modified or adapted from various sources (Appendix 1).

We used a four-item scale to measure participants' ICT self-efficacy. The general concept of self-efficacy was based on Schwarzer and Jerusalem (1995); Teo (2009) survey, and the facets of ICT self-efficacy were based on the scales used in the studies of Van Acker et al. (2011) and Aesaert and van Braak (2014). In this research, blended learning-related ICT self-efficacy in the use of computers, online platforms, software, and online teaching resources was assessed.

Organizational support for blended learning was measured with four items in this research. The items were modified based on Eisenberger et al. (1986) and Eisenberger et al. (1997) for the concept of perceived organizational support and Garrison and Kanuka (2004) and Zhao and Song (2021) for organizational issues of blended learning.

The three items for the measurement of attitudes toward blended learning were adopted from the subscales used by Van Acker et al. (2011) and Chan and Chen (2014).

The measure for the use of blended learning was based on Garrison and Kanuka (2004) definition. It contains six items. Items related to online teaching were built with reference to Shea et al. (2006) concept of teaching presence and Coates (2007) model of online and general campus-based student engagement.

### Data Collection and Data Analysis

We conducted the questionnaire from 26 January to 28 March 2022 and adopted snowball sampling for data collection. Before beginning to fill out the questionnaire, teachers were informed of the purpose of the questionnaire, and data collection was strictly in accordance with the rules and guidelines applicable to ethical research practices involving human participants. The online questionnaire platform Wenjuanxing and English teachers' working WeChat groups were used to collect data and recruit participants.

Partial least squares structural equation modeling (PLS-SEM) was applied in the data analysis in this research. According to Garson (2016), PLS-SEM works well for exploratory modeling analysis. In terms of reliability and validity, it allows researchers to measure models and analyze the associations among theoretical constructs of the models (Hair et al., 2013). The present study is an exploratory one based on previous studies, and some items in the questionnaire were self-designed. In this regard, PLS-SEM is suitable for data analysis in this study. We employed SmartPLS 3.0 to test the measurement models and the structural model designed. In addition, initial descriptive statistics and correlation analyses were conducted with SPSS 24.0.

## Results

### Descriptive Analysis

Table 2 presents the value of means, SDs, and values of skewness and kurtosis of four constructs of the research model. All means are above the medium level of 3.00, indicating that English teachers demonstrated a relatively high-level ICT self-efficacy, organizational support for blended learning, attitudes toward blended learning, and the use of blended learning. In particular, attitudes toward blended learning with the highest mean value (M = 3.74) demonstrated that English teachers had a strong positive attitude toward blended teaching. The SDs are from 0.62 to 0.71, indicating narrow spreads around the means. The values for skewness and kurtosis are lower than 1.00, suggesting that the data for all constructs are normally distributed.

**Table 2:** Descriptive statistics of the study constructs.

**Construct**	**Item**	**Mean**	**Standard deviation**	**Skewness**	**Kurtosis**
ICTSE	4	3.21	0.62	0.14	−0.08
OSBL	4	3.57	0.65	−0.03	−0.18
ABL	3	3.74	0.71	−0.39	0.36
UBL	6	3.48	0.70	−0.31	0.33

*N = 562; ICTSE, ICT self-efficacy; OSBL, organizational support for blended learning; ABL, attitude toward blended learning; UBL, the use of blended learning*.

### Analysis of the Measurement Model

We assessed the measurement model by testing the internal consistency, convergent validity, and discriminant validity.

Item reliability, composite reliability (CR), and the average variance extracted (AVE) of each construct were assessed in this research for internal consistency and convergent validity of the measurement items based on Fornell and Larcker (1981) suggestion. The factor loadings of all the items in the measurement model in this research ranged from 0.73 to 0.95 (Table 3). All of them are above 0.70, the recommended threshold by Gefen et al. (2000) and Hair et al. (2006), thus indicating convergent validity for items of all constructs. According to Nunnally and Bernstein (1994), a value of 0.70 or higher is adequate for composite reliability. The CR value of each construct in this research ranged from 0.88 to 0.92, far above the recommended value of 0.70. Cronbach's alpha and Dillon–Goldstein's rho for the four constructs in this research ranged from 0.83 to 0.93 and 0.84 to 0.94, respectively, congruent with the acceptable criterion (Nunnally, 1968). AVE is an indicator of convergent validity that can be used to measure the overall amount of variance attributed to the construct (Fornell and Larcker, 1981). An AVE value of 0.50 or higher could be considered adequate for the convergent validity of a construct (Fornell and Larcker, 1981). As shown in Table 3, the convergent validity for all constructs of the research model is adequate (AVE ranged from 0.66 to 0.87). As shown in Table 4, square roots of the average variance extracted are greater than the corresponding off-diagonal correlations, thus the discriminant validity is achieved (Fornell and Larcker, 1981).

**Table 3:** Results of the measurement model.

**Latent variable**	**Item**	**Factor loading**	**Cronbach's alpha**	**rho_A**	**CR**	**AVE**
ICTSE	I1	0.73	0.924	0.924	0.952	0.868
	I2	0.87				
	I3	0.83				
	I4	0.81				
OSBL	O1	0.87	0.826	0.842	0.884	0.657
	O2	0.86				
	O3	0.91				
	O4	0.82				
ABL	A1	0.94	0.884	0.887	0.92	0.742
	A2	0.95				
	A3	0.91				
UBL	U1	0.90	0.939	0.941	0.951	0.766
	U2	0.87				
	U3	0.92				
	U4	0.88				
	U5	0.82				
	U6	0.86				

*CR, Composite reliability; AVE, Average variance extracted*.

**Table 4:** Discriminant validity for the measurement model.

**Construct**	**ABL**	**ICTSE**	**OSBL**	**UBL**
ABL	**0.932**			
ICTSE	0.395**	**0.811**		
OSBL	0.528**	0.357**	**0.862**	
UBL	0.631**	0.555**	0.549**	**0.875**

**p < 0.01.

*Diagonals shown in bold are square roots of the average variance extracted from observed items*.

*Off-diagonal are correlations between constructs*.

The testing results for the internal consistency, convergent validity, and discriminant validity are satisfactory for all four constructs at both the item and construct levels, suggesting that the constructs of the research model in this study are suitable for further analyses.

### Analysis of the Structural Model

**Figure 2:**
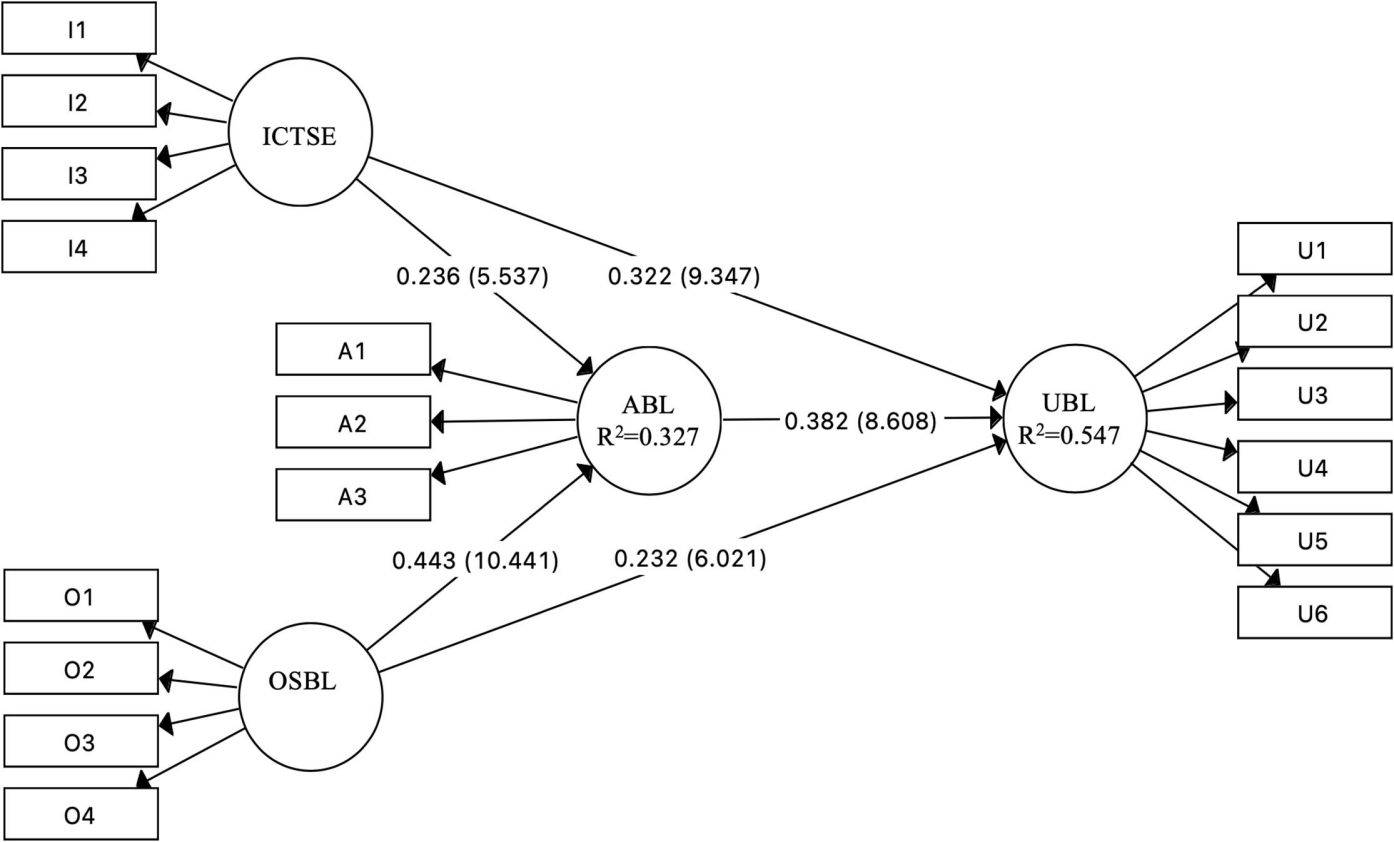
PLS-SEM for predicting the use of blended learning (the values in parentheses are T values).

The significance of the path coefficients and R square values were checked to examine the structural model and test the hypotheses proposed in this research. The bootstrapping procedure was used to assess the significance levels of the path coefficients, according to Hair et al. (2017). It was run with 5,000 samples to gain precise estimates. The results obtained are shown in Table 5 and Figure 2.

**Table 5:** Hypothesis testing.

**Hypotheses**	**Path coefficient**	**Standard deviation**	**T value**	***p* Value**	**Results**
Direct effects					
H1: ABL → UBL	0.38	0.04	8.61	0.00	Supported
H2: ICTSE → ABL	0.24	0.04	5.55	0.00	Supported
H3: ICTSE → UBL	0.32	0.03	9.35	0.00	Supported
H4: OSBL → ABL	0.44	0.04	10.36	0.00	Supported
H5: OSBL → UBL	0.23	0.04	6.07	0.00	Supported
Indirect effects					
H6: ICTSE → ABL → UBL	0.09	0.02	4.39	0.00	Supported
H7: OSBL → ABL → UBL	0.17	0.03	6.91	0.00	Supported

As expected, attitude toward blended learning is positively and directly related to teachers' behavior in using blended learning (b = 0.38, *p* < 0.01), therefore supporting H1. It was found that ICT self-efficacy could significantly and directly influence attitudes toward blended learning (b = 0.24, *p* < 0.01) and the use of blended learning (b = 0.32, *p* < 0.01), supporting H2 and H3. Likewise, organizational support for blended learning could significantly and directly influence attitudes toward blended learning (b = 0.44, *p* < 0.01) and the use of blended learning (b = 0.23, *p* < 0.01), supporting H4 and H5. In addition, mediated by attitudes toward blended learning, ICT self-efficacy is positively and indirectly related to the use of blended learning (b = 0.09, *p* < 0.01), and organizational support for blended learning is positively and indirectly related to the use of blended learning (b = 0.17, *p* < 0.01), supporting H6 and H7, respectively.

R square was used in this research to test the predictive relevance of the structural model. According to Chin (1998), the values of 0.02, 0.03, and 0.26 for R square can be interpreted as effect sizes of small, medium, and large, respectively. As shown in Figure 2 and Table 6, R-squared values for endogenous variables are 0.327 and 0.547, indicating high levels of predictive power. Specifically, 32.7% of the variance in attitudes toward blended learning can be predicted by ICT self-efficacy and organizational support for blended learning. In addition, 54.7% of the variance in the use of blended learning can be predicted by attitudes toward blended learning, ICT self-efficacy, and organizational support for blended learning.

**Table 6:** Predictive relevance and effect size.

**Endogenous variable**	**R Square**	**Predictive variable**	**F Square**
ABL	0.327	ICTSE	0.072
		OSBL	0.255
UBL	0.547	ABL	0.217
		ICTSE	0.186
		OSBL	0.083

In addition to the use of R square in assessing the predictive power of the research model, effect size F square was also used to test the predictive relevance. Effect size F square indicates the contributions of predictive variables to endogenous variables, and the values of 0.02, 0.15, and 0.35 demonstrate small, medium, and large effects, respectively (Hair et al., 2014a,b). As shown in Table 6, ICT self-efficacy had medium effects on attitudes toward blended learning (F^2^ = 0.072) and the use of blended learning (F^2^ = 0.186); organizational support for blended learning had medium effects on attitudes toward blended learning (F^2^ = 0.255) and the use of blended learning (F^2^ = 0.083); and attitudes toward blended learning had a medium effect on the use of blended learning (F^2^ = 0.217).

## Discussion

Based on survey data of 562 English teachers in basic education in a blended learning environment created by repeated lockdowns during the COVID-19 pandemic, this study explored the influence of ITC self-efficacy, organizational support for blended learning, and attitudes toward blended learning on the use of blended learning.

This study found that ICT self-efficacy is positively and directly related to the use of blended learning. While there is currently no shortage of evidence for a link between self-efficacy and use behavior and evidence that ICT self-efficacy is important for both students' learning processes and teachers' instruction (Aesaert and van Braak, 2014; Moreira-Fontán et al., 2019), the relationship between ICT self-efficacy and teachers' use of blended learning remains unclear. Previously, only Yeop et al. (2019) research based on 720 Malaysian teachers showed that the implementation of ICT policies was related to teachers' use of blended learning. This study more precisely demonstrates that there is an effective direct link between ICT self-efficacy and teachers' use of blended learning. It is found that teachers with greater ICT self-efficacy are more likely to perform better with using blended learning.

In addition, our research found that organizational support for blended learning can be used as a direct predictor of teachers' use of blended learning. In this research, we built a construct to assess teachers' perceived organizational support for blended learning, and the direct path coefficient between them indicated that they are positively related to each other. This finding is in line with implications proposed by Garrison and Kanuka (2004) and Zhao and Song (2021), although with more concrete evidence.

Furthermore, this study also proved that attitudes toward blended learning can directly influence teachers' use of blended learning and that attitudes play an important role as the mediator for the relationship between ICT efficacy and the use of blended learning and between organizational support for blended learning and the use of blended learning. The attitude was used as an antecedent for actual behavior in TAM-based research, with the mediation of behavioral intention. In this study, we designed a research model with a direct path between attitude and behavior and found that teachers with more positive attitudes were more likely to use blended learning at a higher level. Both ICT self-efficacy and organizational support for blended learning are directly and positively related to attitudes toward blended learning in this research. In addition, attitudes toward blended learning mediated both the relationship between ICT self-efficacy and the use of blended learning and that between the organizational support for blended learning and the use of blended learning. Surprisingly, we found that organizational support for blended learning contributes more to the use of blended learning both directly and indirectly with the mediation of attitudes toward blended learning.

Based on the above findings, this study has several implications for school administrators and teachers. Given how important ICT self-efficacy and perceived support for blended learning are to teachers in a blended-learning environment and that teachers are reluctant to integrate it into their teaching practices (Jimoyiannis and Komis, 2007; Yang and Huang, 2008), administrators must provide adequate support for teachers' training of ICT, hardware and software, experience sharing opportunities, incentives and support policies related to the implementation of blended learning for the teachers. Teachers should work with administrators to overcome the resistance or fear of ICT and improve their ICT self-efficacy and ICT capabilities through more sharing and learning activities to achieve more efficient use of blended learning.

This study has the following limitations. First, the participants involved are all English teachers at the basic education stage in Guangdong Province, China. Therefore, its generalizability to teachers of other subjects, stages, and places are yet to be confirmed. Second, the research model designed in this study is relatively simple, leaving much of the variance unexplained. In addition, this research was conducted in the environment of repeated lockdowns during the COVID-19 pandemic, when some teachers were forced to use blended learning; hence, the effectiveness of the research model in the post-pandemic period needs further confirmation. For further research, teachers from other subjects, stages, and places can be studied, and the invariance of those different types of teachers for this research model can be examined. In addition, more variables can be added to the research model. For example, verified predictors for ICT self-efficacy, such as autonomous learning and experience with ICT (Hatlevik et al., 2018), and other predictors for the use of blended learning can be added to improve the explanation of the variation in teachers' use of blended learning.

## Data Availability Statement

The raw data supporting the conclusions of this article will be made available by the authors, without undue reservation.

## Ethics Statement

The studies involving human participants were reviewed and approved by Ethics Committee of School of Foreign Studies, Shaoguan University. The patients/participants provided their written informed consent to participate in this study.

## Author Contributions

LY: conceptualization, methodology, data analysis, and writing–original draft. MK: data collection. SL: resources, and writing–review and editing. All authors have read and agreed to the published version of the manuscript.

## Funding

This project was supported by four research programs in China, including Hunan Provincial Educational Teaching Reform Project (Grant No. HNJG-2021-1149), Program of Center for Translation Studies of Guangdong University of Foreign Studies (Grant No. CTS202108), Scientific Research Program of Education Department of Hunan Province (Grant No. 21B0834), and College Student Innovation and Entrepreneurship Training Program of Guangdong Province (Grant No. S202110576054).

## Conflict of Interest

The authors declare that the research was conducted in the absence of any commercial or financial relationships that could be construed as a potential conflict of interest.

## Publisher's Note

All claims expressed in this article are solely those of the authors and do not necessarily represent those of their affiliated organizations, or those of the publisher, the editors and the reviewers. Any product that may be evaluated in this article, or claim that may be made by its manufacturer, is not guaranteed or endorsed by the publisher.

## Appendix 1

**Appendix:** List of constructs and corresponding items.

**Construct (references)**	**Item**	
ICT self-efficacy (Schwarzer and Jerusalem, 1995; Van Acker et al., 2011; Aesaert and van Braak, 2014)	I1	I think I can use computers well for teaching.
	I2	I think I can make good use of the online teaching platform.
	I3	I think I can make good use of software to record online lessons.
	I4	I think I can make good use of online teaching resources.
Organizational support of blended learning (Eisenberger et al., 1986; Eisenberger et al., 1997; Garrison and Kanuka, 2004, Zhao and Song, 2021)	O1	I can perceive the administration's support for blended learning.
	O2	The network environment provided by the school can provide effective support for blended learning.
	O3	I can perceive the school's support for blended learning.
	O4	I can perceive the support of my colleagues at school for blended learning
Attitude towards blended learning (Van Acker et al., 2011; Chan and Chen, 2014)	A1	I think blended teaching makes it easier for me to collect and grade student submissions.
	A2	I think blended teaching allows me to quantify student learning.
	A3	I think blended teaching can improve teaching efficiency.
Use of blended learning (Garrison and Kanuka, 2004; Shea et al., 2006; Coates, 2007) '	U1	I use online teaching methods to match the learning tasks of offline courses.
	U2	I use the online method to assign study tasks for students to preview or review.
	U3	I integrate online and offline teaching to guide students to correctly understand the learning content.
	U4	In the online class, I give detailed explanations to students on problems.
	U5	I organize activities such as online group discussions to help students learn.
	U6	I integrate online and offline teaching to carry out personalized teaching.
